# Therapeutic treatment with the anti-inflammatory drug candidate MW151 may partially reduce memory impairment and normalizes hippocampal metabolic markers in a mouse model of comorbid amyloid and vascular pathology

**DOI:** 10.1371/journal.pone.0262474

**Published:** 2022-01-26

**Authors:** David J. Braun, David K. Powell, Christopher J. McLouth, Saktimayee M. Roy, D. Martin Watterson, Linda J. Van Eldik

**Affiliations:** 1 Department of Neuroscience, University of Kentucky, Lexington, Kentucky, United States of America; 2 Sanders-Brown Center on Aging, University of Kentucky, Lexington, Kentucky, United States of America; 3 Magnetic Resonance Imaging and Spectroscopy Center, University of Kentucky, Lexington, Kentucky, United States of America; 4 Department of Behavioral Science, University of Kentucky, Lexington, Kentucky, United States of America; 5 Department of Pharmacology, Northwestern University, Chicago, Illinois, United States of America; Nathan S Kline Institute, UNITED STATES

## Abstract

Alzheimer’s disease (AD) is the leading cause of dementia in the elderly, but therapeutic options are lacking. Despite long being able to effectively treat the ill-effects of pathology present in various rodent models of AD, translation of these strategies to the clinic has so far been disappointing. One potential contributor to this situation is the fact that the vast majority of AD patients have other dementia-contributing comorbid pathologies, the most common of which are vascular in nature. This situation is modeled relatively infrequently in basic AD research, and almost never in preclinical studies. As part of our efforts to develop small molecule, anti-inflammatory therapeutics for neurological injury and disease, we have recently been exploring potentially promising treatments in preclinical multi-morbidity contexts. In the present study, we generated a mouse model of mixed amyloid and hyperhomocysteinemia (HHcy) pathology in which to test the efficacy of one of our anti-inflammatory compounds, MW151. HHcy can cause cerebrovascular damage and is an independent risk factor for both AD dementia and vascular contributions to cognitive impairment and dementia. We found that MW151 was able to partially rescue hippocampal-dependent spatial memory and learning deficits in this comorbidity context, and further, that the benefit is associated with a normalization of hippocampal metabolites detectable via magnetic resonance spectroscopy. These findings provide evidence that MW151 in particular, and potentially anti-inflammatory treatment more generally, may be beneficial in AD patients with comorbid vascular pathology.

## Introduction

Aging-related dementia is a growing public health concern, exacting large social and economic costs that are increasing in parallel with an aging population [1]. The primary driver of this dementia is Alzheimer’s disease (AD), which is clinically diagnosed (probable AD) by the gradual onset of progressive cognitive or behavioral impairments, below-criterion performance in various standardized neurological measurements, absence of a clear alternative explanation for the mental changes and, increasingly, specific biomarker-based changes [2]. Confirmation of an AD diagnosis is made at autopsy by assessment of canonical amyloid beta (Aβ) peptide accumulations (plaques) and intraneuronal aggregations of tau protein (tangles) [3]. Over time it has become apparent that patients with pure AD pathology—*i*.*e*., only plaques and tangles—are the exception rather than the rule. Indeed, in a recent review [4], the authors found that only about 3% of patients with a probable AD diagnosis had solely AD-type neuropathology at autopsy. The most common comorbidity by far is cerebrovascular pathology, present in about 75% of cases and consisting primarily of infarcts and/or small vessel disease changes. In this context, the near-perfect failure rate of preclinically-promising AD therapeutics [5] implicate one particular concern: inadequate modeling of the dementia-contributing pathologies commonly present in AD patient populations. From a translational standpoint, the demonstration that a particular treatment is preclinically efficacious in the context of pure amyloid or tau pathology appears to be an insufficient predictor of future clinical success. The present study therefore aims to test the efficacy of a brain-penetrant small-molecule anti-inflammatory therapeutic, MW151, in the context of comorbid amyloid and vascular pathology.

MW151 is currently in Phase 1 clinical safety testing for AD (ClinicalTrials.gov identifier: NCT04120233) based upon benefit found across diverse neurological injury and disease models. In addition to ameliorating neuropathology in two amyloid-based models of “pure AD” pathology [6, 7], MW151 also provides therapeutic benefit in models of traumatic brain injury (TBI) [8–10], seizure disorders [11–13], radiation-induced cognitive impairment [14] and multiple sclerosis [15]. Importantly, MW151 has shown efficacy in two dual-pathology contexts: one combining TBI and A® pathology [16], and one combining TBI and electroconvulsive shock [17]. Interestingly, there is evidence that the enhanced risk for dementia attributable to TBI may be mediated primarily through vascular dysfunction [18]. Given the abundance of vascular pathology in AD patients, we decided to directly test whether MW151 could also provide benefit in the more clinically common context of comorbid A® and vascular pathology.

For this study we generated a mouse model of mixed dementia (MD) by administering 8 weeks of a B-vitamin deficient and methionine-supplemented diet to amyloid overexpressing mice. Previous work has shown that 8 weeks of this diet is sufficient to cause moderate to severe hyperhomocysteinemia (HHcy), blood-brain barrier dysfunction and microhemorrhages, glial activation, cerebral blood flow (CBF) deficits, and an array of changes in vascular-associated genes [19, 20]. We have also shown that 8 weeks of HHcy diet in an amyloid context can enhance plaque burden, potentiate proinflammatory cytokine production, and alter microglial-associated gene expression [21], consistent with what has been previously reported with longer HHcy time courses in different models [22–24]. To avoid potential confounds from acute systemic effects of severe vitamin deficiencies, we included a 2-week dietary recovery period following 8-week HHcy. After recovery, mice were treated with saline or MW151 for 14 days and underwent behavioral testing or magnetic resonance imaging (MRI) during the final week of treatment.

We found that MW151 partially rescued hippocampal-dependent behavioral deficits in this MD model, corresponding with a normalization of hippocampal levels of the neuronal integrity marker N-acetylaspartate (NAA) and the neuropeptide N-acetylaspartylglutamate (NAAG). These changes occurred alongside the expected anti-inflammatory signature of MW151: a reduction in proinflammatory cytokines and astrocyte activation. This study provides evidence that MW151 might provide functional benefit in AD patients with comorbid vascular pathology, and also indicates that magnetic resonance spectroscopy (MRS) endpoints may be useful as non-invasive surrogate markers of efficacy for later-stage clinical trials of this drug candidate.

## Materials and methods

### Animals and experimental design

#### Model of mixed dementia (MD)

All studies involving vertebrate animals were approved by the University of Kentucky Institutional Animal Care and Use Committee (IACUC), under protocol 2012–0932. Isoflurane (1–2%) was used to maintain anesthesia during neuroimaging; 5% isoflurane was used during euthanasia by exsanguination.

The MD mice were generated by placing amyloid overexpressing mice on a transient HHcy-inducing diet. Male and female B6.Cg-Tg(APPswe,PSEN1dE9)85Dbo/Mmjax (MMRRC No: 34832-JAX) transgenic AD model mice (APPswe/PS1dE9) were used for this study, along with noncarrier wildtype (WT) littermates. All mice were maintained on a congenic C57BL/6J background. Carrier mice express a chimeric humanized amyloid precursor protein (APP) with the Swedish mutation, and a mutant human presenilin 1 (PS1) missing exon 9, resulting in progressive accumulation of A® peptide, plaque deposition, concomitant neuroinflammatory changes, and eventual behavioral impairment [25–27]. To induce HHcy, mice were placed on a custom diet (Envigo Teklad TD.97345) for 8 weeks beginning at about 34 weeks of age (mean = 33.8, standard deviation = 0.7). The diet lacks vitamins B_6_, B_9_ (folate), and B_12_ and has excess methionine [20]. Control mice were given 8 weeks of a nutritionally matched custom control diet with normal methionine and B-vitamin levels (TD.01636). See **S1 Table** for details of both diets. All mice were subsequently returned to standard nutritionally complete rodent chow (TD.2918) for a 2-week recovery period, followed by 2-weeks of once daily intraperitoneal injections of either 5 mg/kg MW151 or an equivalent volume of saline vehicle. Throughout the experiments mice were housed 1–5 animals per cage (503.22 usable cm^2^), maintained in a room at 23°C ± 2°C with a 14/10 hour light/dark cycle beginning at 6:00 AM, and had *ad libitum* access to food and water. Environmental enrichment included a 5x5 cm cotton Nestlet (Ancare, Bellmore, NY, USA), paper shredding, a Backless Shack mouse house (Shepherd Specialty Papers), and ½ x ½ x 2” aspen chew sticks (Lomir Biomedical Inc).

#### Overall experimental design

Two separate cohorts were generated for these studies. Cohort 1 was tested in a behavioral battery and cohort 2 underwent magnetic resonance imaging (MRI). Both cohorts consisted of the same three primary experimental groups: WT mice given control diet and administered IP saline injections (WT ctrl), APP/PS1 mice given HHcy diet and administered IP saline injections (MD saline), and APP/PS1 mice given HHcy diet and administered IP MW151 injections (MD MW151). An additional group was included in the behavioral cohort to validate the presence of persistent cognitive deficits despite reversal of HHcy: WT mice given HHcy diet and administered saline (WT HHcy) (see **S1 Fig**). Verification of vascular injury by Prussian blue (PB) staining in the MD model mice is contained in **S2 Fig**, consistent with what has previously been reported as a result of this diet [20, 23]. Mice in the behavioral cohort were tested during days 7–14 of dosing (see behavioral battery section below). Mice in the MRI group were imaged between day 12 and 14 of dosing. Injections were performed after the conclusion of the behavioral or neuroimaging assays for that day. All mice were sacrificed within 14–16 hours of receiving the final dose of MW151 at about 46 weeks of age. Tissue was harvested from the behavioral battery cohort for biochemical and histological analyses to avoid potential confounding effects of extended isoflurane administration in the neuroimaging group [28].

### Experimental cohort 1—Behavioral testing

#### Behavioral battery design

**Fig 1:**
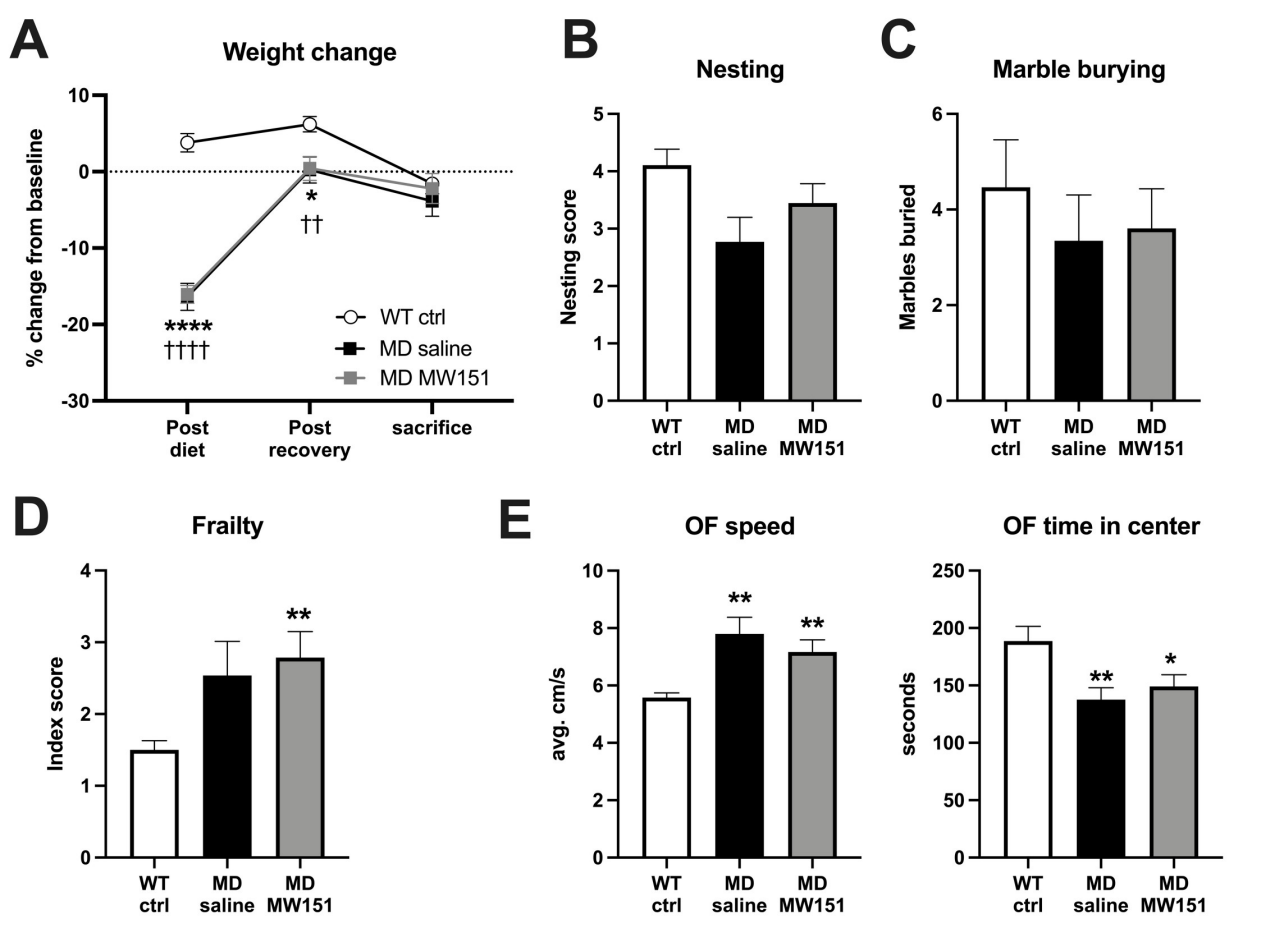
MD mice show altered non-cognitive behaviors versus WT control, unaffected by treatment with MW151. (**A**) MD mice in both treatment groups lost significant weight on HHcy diet, which was recovered by time of sacrifice and unaffected by MW151 treatment. Results of two-way mixed measures ANOVA revealed a significant effect of time (*F*(1.693, 86.34) = 178.0, *p* < .0001), group (*F*(3, 51) = 13.02, *p* < .0001), and a time by group interaction (*F*(6, 102) = 24.02, *p* < .0001). ****p < .001, *p < .05 for the MD saline mice versus WT ctrl comparisons and ††††p < .001, ††p < .01 for MD MW151 versus WT ctrl, Sidak’s posthoc tests. (**B**) No significant differences between groups are observed in the nesting assay, despite overall significance in the Kruskal-Wallis test (*H*(4) = 9.833, *p* = 0.020). (**C**) No differences in the number of marbles buried are observed between any of the groups (*F*(3, 51) = 0.3992, *p* = 0.75). (**D**) There was a significant effect in the Frailty index scores (*H*(4) = 10.78, *p* = 0.0129), with the MD MW151 mice found to have a significantly higher score than WT ctrl, **p < .01, Dunn’s post hoc test. (**E**) Open field speed and time in center are shown, where the MD saline mice have a higher average speed than WT ctrl, and also spend less time in the center of the arena. MW151 does not alter either of these parameters. Results of one-way ANOVAs showed significant overall differences in average OF speed (*F*(3, 51) = 10.89, *p* < .0001) and OF time in center (*F*(3, 51) = 4.038, *p* = 0.0119), **p < .01, *p < .05 versus WT ctrl group, Holm-Sidak post-hoc comparisons.

**Fig 2:**
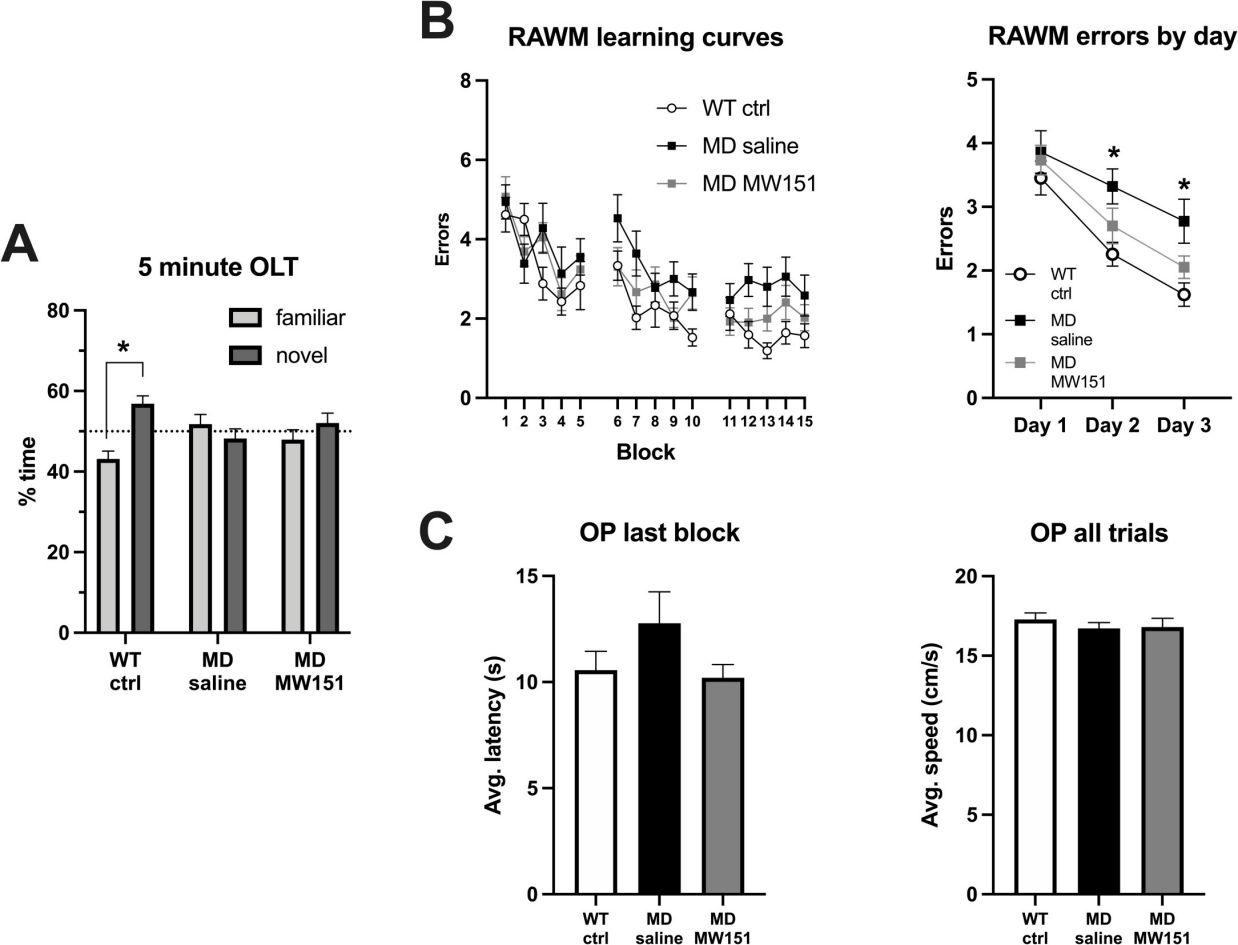
MW151 treatment in MD mice has no effect on OLT performance but may reduce errors in the RAWM. (**A**) Neither the MD saline nor the MD MW151 groups spent significantly more time on average exploring the object in the novel versus the familiar location, in contrast to the WT ctrl group which did show a significant preference for the object in the novel location: multiple paired t-tests with Holm-Sidak adjustment (*T*(13) = 3.522, *p* = 0.0149) *p < .05. (**B**) Learning curves of RAWM errors by block (3 consecutive trials) are shown for the WT ctrl and MD mice in the left panel. Errors are collapsed into average per day for statistical analysis in the right panel. Two-way mixed measures ANOVA showed a significant effect of day (*F*(1.955, 97.73) = 41.08, *p* < .0001) and group (*F*(3, 50) = 4.088, *p* = 0.0113). The MD saline mice (n = 12) committed significantly more errors than WT ctrl on both day 2 and 3 of testing, *p < .05 versus WT ctrl group, Tukey post-hoc comparisons. In contrast, MD MW151 mice did not commit statistically more errors than WT ctrl on any day. There was no significant difference between the MD MW151 and MD saline groups on any of the three days. (**C**) Results of one-way ANOVAs for average latency to platform in the final block (*F*(3, 50) = 1.076, *p* = .368) and average swim speed (*F*(3, 50) = 0.278, *p* = 0.841) indicated no statistical difference between any groups.

This experiment consisted of 14 WT ctrl mice (10F/4M), 13 MD saline mice (7F/6M), and 14 MD MW151 mice (7F/7M). There were 14 mice in the WT HHcy group included for verification (5F/9M) of persistent HHcy-induced cognitive deficits. Mice in all 4 groups were run and analyzed together statistically, but for purposes of clarity only the primary comparisons of interest (WT ctrl, MD saline, and MD MW151 groups) are shown in **Figs 1 and 2**. To facilitate acclimatization to the experimenter, mice were gently handled during weekly diet changes and weigh-ins using the cupping method [29], as well as for 4 non-consecutive days during the two-week diet recovery period. The battery was carried out over 7 days beginning on day 8 of IP injections. All testing was performed in designated behavioral testing rooms, with mice transported in their home cages and allowed to habituate for one hour each day prior to testing. On dosing day 8, mice underwent open field (OF) testing in the morning, followed by the frailty assay in the afternoon. On the morning of day 9, mice underwent the short-term object location test (OLT) in the same arena as open field. On days 11–13 mice underwent testing in the 6-arm radial arm water maze (RAWM). In the morning of day 14, mice were tested in the open pool (OP) followed by the marble burying task in the afternoon. In the evening, mice were separated and singly housed in a cage with a fresh nestlet for the overnight nesting assay. The following morning mice were humanely euthanized for tissue harvest and their nests were scored by blinded staff. Automated tracking was performed with Noldus EthoVision XT 10 (Noldus, Leesburg, Virginia, USA) for OF, OLT, and RAWM. All trials were recorded and monitored in real-time during all trials for all tests. In the event of tracking failure, the videos were manually re-scored by a blinded lab member.

#### Open field (OF)

The open field test was performed as described previously [30]. The testing apparatus consisted of four adjacent 40 cm x 40 cm arenas with overhead lighting set to approximately 20 lux. An individual mouse was placed into each arena to allow simultaneous testing of up to four mice at a time, with each open field trial lasting 15 minutes. Infrared cameras were used to enable body center-point tracking in the low light, and the measurement of total distance traveled, average speed of movement, and percent time in the center zone of the arena (20 x 20 cm area) was performed automatically.

#### Frailty assay

Mice were tested according to [31]. Briefly, each mouse was assessed by a blinded observer across 27 aging-related parameters and graded based on presence and severity of each. A score of 0 indicated normal/absence of age-associated changes, 0.5 indicated modest degree of the trait, and 1 indicated severe degree. The final scores for each trait were then summed to generate a frailty index score, with a higher score indicating increased age-associated changes. Core body temperature was also measured with a rectal probe. See **S2 Table** for traits and grading criteria used.

#### 5-minute object location test (OLT)

This test was performed in the same apparatus as the open field assay, but with two identical Lego® pyramids placed in the top left and top right corners of the box, 5 cm distant from the edges of the arena. Mice underwent a familiarization phase wherein they were given 10 minutes to freely explore both objects, followed by a 5-minute intertrial interval spent in a clean and empty holding cage. During this time, both objects were replaced with identical but clean objects, but the upper right corner object moved instead to the lower right corner. Mice were then returned to their assigned arena for the testing phase, wherein they were given 5 minutes to freely explore both objects. Detection of nose-point within 1 cm of the object counted as exploration of that object.

#### Six-arm radial arm water maze (RAWM)

RAWM was run according to the protocol previously described [19]. Briefly, the maze consisted of six arms in a circular tank filled with water containing non-toxic white tempera paint. The tank was enclosed by black curtains arranged in a square, with geometric extra-maze visual cues affixed to three of four curtain sides. A circular platform was placed at the end of one of the arms, approximately 1–2 cm below the water’s surface to provide an escape. Animals were split into two groups and tested in a back-to-back 3-day paradigm. Each day consisted of 15 trials (45 trials total), with the first 12 trials on day 1 alternating between hidden and visible (flagged) platform. The platform was hidden for all trials after the first 12. Trials were run in two blocks of 6 and one block of 3 each day. Mice were run in groups of 5–8 at a time, alternating by block to provide time to rest. The goal arm was fixed for each mouse but varied between animals, and each mouse started from a different arm between trials. The start arm sequence was randomized in reference to the goal arm, but with a pattern consistent for all mice. Errors were defined as entry from the pool center into any non-goal arm, or 15 seconds in any location without movement to a new zone. Entry to a new zone was defined in the software as movement of the center point of the mouse ≥ 5 cm past the dividing line between zones. To visualize learning curves, groups of 3 consecutive trials were averaged into blocks, for a total of 5 blocks per day. For statistical analysis by ANOVA, total errors were averaged by day.

#### Open pool (OP)

The day following RAWM, all mice underwent testing for swimming/motivational or visual impairments in the open pool [32]. The arms and visual cues were removed from the testing arena, and the platform raised to ~0.5 cm above the water surface. Mice received 10 trials each, with the platform raised and flagged for all trials. Each mouse was assigned a starting quadrant and the platform position alternated between the 3 remaining quadrants between trials. Time to find the platform from the starting quadrant was measured for each trial. Criterion for impairment was defined as an average latency to platform of > 20 seconds for the final block (last 3 trials). One female mouse from the MD saline group reached the impairment criterion (49.6 s) and was excluded from the RAWM and OP analyses.

#### Marble burying assay

The marble burying test was performed with a slight modification of the protocol previously published [33]. Each mouse was tested in a new housing cage without food or water, filled to a height of 5 cm with fresh bedding material and 12 glass marbles (1.4 cm diameter) evenly spaced in a grid pattern. After exploring the cage for 30 minutes the mouse was carefully removed and a picture of the cage bottom taken from directly overhead. Two blinded scorers counted the number of buried marbles (defined as < 50% of the marble surface still visible) and their scores were averaged.

#### Nesting assay

Nesting assay was performed as previously described [19, 34]. Briefly, mice were singly housed overnight in a new cage with no enrichment save for bedding and a new Nestlet. Two observers blinded to the experimental conditions scored the quality of the nests according to a semi-quantitative 5-point scale: (1)–nestlet not noticeably touched, >90% intact; (2)–nestlet partially torn up, 50–90% remaining intact; (3)–shredded >50% but without identifiable nesting site and bedding spread around cage; (4)–identifiable but flat nest, >90% of the nestlet torn up with the material gathered within one quarter of the cage floor area; (5)–a near perfect nest where >90% of the nestlet is torn up, the nest is crater shaped, and walls are higher than the mouse body for at least half of the circumference. Scores were assigned for each nest in half-point increments and averaged between the observers to generate an average nesting score.

#### Brain tissue harvest and plasma homocysteine measurement

To avoid potential confounding effects of extended isoflurane administration during neuroimaging on endpoints of interest, tissue was harvested from the mice that underwent behavioral testing as previously reported [19, 21]. Briefly, mice were anesthetized with 5% isoflurane, blood was collected via cardiac puncture and mice were transcardially perfused over 5 minutes with 50 mL cold 1x PBS before brain removal and dissection. Whole blood was separated by centrifugation in EDTA tubes (Greiner Bio-One, #454428) at 2000xg for 20 minutes at room temperature. Plasma was collected, flash frozen in liquid nitrogen and stored at –80°C until processing for homocysteine measurement. Plasma samples from a randomly chosen subset of 5 mice from each group were diluted 1:5 in ARCHITECT Multi-Assay Manual Diluent and analyzed on an ARCHITECT i2000SR instrument at the University of Kentucky Clinical Laboratory. The brain was cut down the midline and the right hemisphere immediately post-fixed in 4% paraformaldehyde for 18-24h at 4°C and cryopreserved in 30% sucrose for a minimum of 48 h at 4°C. Fixed samples were cut into 30 μm sections and stored in cryoprotectant solution (50% 1X PBS, 25% ethylene glycol, 25% glycerol) at -20°C until staining. The hippocampus was removed from the left hemisphere, flash frozen in liquid nitrogen, and stored at -80°C until processing for cytokine measurement.

#### Immunofluorescent measurement of GFAP and Thioflavin S-positive amyloid plaques

Six to eight free-floating sections spaced approximately 300 microns apart through the dorsal hippocampus were used for each animal. Sections were blocked with 10% goat serum (Lampire Biological Laboratories, #7332500) and 0.2% Triton X-100 in PBS. Rat anti-GFAP antibody (Invitrogen #13–0300) was diluted 1:3000 in PBS with 3% normal goat serum and 0.2% Triton X-100. Sections were incubated in primary antibody for 14–16 hours at 4°C before a 2-hour room temperature incubation with an Alexa633-conjugated anti-rat secondary antibody (Invitrogen #A-21094, 1:1000). Sections were mounted, partially dried, and treated for 5 minutes with a 1% Thioflavin S (Sigma #1892) solution in water, followed by two 5-minute washes with 80% ethanol. They then underwent a 5-min incubation with 1x TrueBlack (VWR, #10119–144) in 70% ethanol to reduce autofluorescence before drying and coverslipping in Vectashield mounting medium with DAPI (Vector Laboratories, #H-1200). Entire slides were imaged on a Zeiss Axio Scan Z1 digital slide scanner at 20x magnification. Hippocampus was outlined manually in HALO (Indica Labs) by a blinded investigator. Algorithm intensity settings were manually thresholded based upon negative control tissue, and the positive pixel algorithm (Area Quantification FL v1.2) was applied to the outlined regions for the GFAP and Thioflavin S channels. For 2 WT ctrl, 3 MD saline, and 4 MD MW151 animals the automated scanner was unable to achieve consistent autofocus across sections, and these slides were therefore not included in the analyses.

#### MesoScale discovery cytokine enzyme-linked immunosorbent assay (ELISA)

Hippocampus dissected from the left hemisphere was homogenized using an Omni Bead Ruptor 24 (Omni International) at a 1:20 weight to volume ratio in PBS lysis buffer: PBS with 1 mM phenylmethylsulfonyl fluoride, 0.5 mM EDTA and 0.2X Halt Protease Inhibitor Cocktail (Thermo Scientific, #87786). Homogenates were centrifuged at 12,000×g for 20 min at 4°C. Supernatants were collected for cytokine measurement using MesoScale Discovery custom mouse V-Plex ELISA kits, with IL-1β, IL-6, TNFα, and CXCL1 run multiplexed as part of the Proinflammatory Panel 1 Mouse Kit (K15048) and run according to manufacturer’s instructions on the QuickPlex SQ 120 reader. Only IL-1β is displayed in the results section as significantly altered in the disease model, but the other cytokines can be viewed in **S3 Fig** and **S1 File**. All samples were incubated for 16–18 hours in the plate at 4°C, shaking at 1000 rpm. Cytokine levels were normalized to the total milligrams (mg) of protein loaded in the sample as determined by BCA Protein Assay (ThermoFisher #23225). Cytokine concentrations were calculated based on standard curves using Discovery Workbench software (Meso Scale Discovery, v4.0). Final values are expressed as the concentration of cytokine measured in the PBS-soluble fraction per mg of total protein loaded per well. One WT ctrl sample was below the limit of detection and one MD saline sample was lost.

### Experimental cohort 2—Magnetic resonance imaging (MRI)

#### Cerebral blood flow (CBF) measurement by pseudo-continuous arterial spin labeling (pCASL)

Mice in the second experiment underwent magnetic resonance imaging at the University of Kentucky Magnetic Resonance Imaging and Spectroscopy Center, with scans performed on a 7 Tesla ClinScan system (Bruker BioSpin, Germany). There were 10 mice per group: 5F/5M WT, 5F/5M MD saline, and 6F/4M MD MW151 group. All mice were anesthetized with 5% isoflurane and transferred to the scanner bed with heating pad, where anesthetic depth was maintained in a ~1.2% isoflurane and air mixture using a nose cone. Imaging was performed after mice reached a stable temperature (35–36°C) and respiratory rate (70–100 breaths/min), which was monitored continuously. A whole-body volume coil was used for transmission and a mouse brain surface coil used for receiving. T2-weighted structural images were acquired with a field of view (FoV) = 23 mm, matrix = 256 x 256; slice thickness = 0.6 mm, 24 slices, repetition time (TR) = 3810 ms, echo time (TE) = 41 ms. For pCASL, interleaved control and labeled images were acquired with a train of 200 Hanning window-shaped radiofrequency pulses of duration = 200 μs, spacing = 200 μs, 25 deg flip angle, max gradient (G_z_) = 90 mT/m, mean G_z_ = 7 mT/m. Two-dimensional multislice spin-echo planar imaging was used with a FoV = 18 mm, matrix = 64 x 64, slice thickness = 1.0 mm, 6 slices, TR = 4,000 ms, TE = 24 ms, with 120 repetitions. A separate normalization image was subsequently acquired with TR = 10,000 ms and 10 repetitions (M_0_). Data processing was performed in a Linux terminal where the tagged signal was subtracted from the untagged and normalized by dividing by M_0_. A single slice at the dorsal hippocampal level was outlined in FSLeyes for all mice and average cerebral blood flow pixel value calculated.

#### Metabolite concentration measurement by proton magnetic resonance spectroscopy (MRS)

After pCASL, a 5.2 x 2.0 x 1.3 mm^3^ voxel was placed around the bilateral hippocampus for water-suppressed MRS measurement: TR = 1500 ms, TE = 135 ms, 400 scans averaged. This was followed by a scan without water suppression, 10 averages. Shim was manually adjusted to get the FWHM below 30 Hz for all animals. Water-suppressed and unsuppressed scans were processed using LCModel with the LCMgui interface [35]. Analyzed metabolite peak values with %SD > 20% were excluded from analyses. If a metabolite was excluded from more than 2 animals of a single group it was not analyzed across groups. Eight metabolites met inclusion criteria: creatine (Cr), phosphocreatine (PCr), lactate (Lac), glutamate (Glu), glutamate and glutamine (Glu+Gln), taurine (Tau), total choline (GPC+PCh), N-acetylaspartate (NAA), and N-acetylaspartate and N-acetylaspartylglutamate (NAA+NAAG). Values for metabolites are expressed as a ratio against the total creatine (Cr+PCr) concentration.

### Data analysis and figure generation

Pre-planned comparisons to examine changes in weight over time by group, and errors by day in the RAWM, were performed using two-way mixed measures ANOVA with the Greenhouse-Geisser correction for sphericity. This was followed by Sidak’s post-hoc testing of each group mean against the WT control group (weight by time) or Tukey’s post-hoc testing to compare every group mean (errors by day in RAWM). The OLT was analyzed by within-group paired t-tests with Holm-Sidak’s correction, comparing the time spent with the object in the novel versus familiar location. Frailty and nesting assays consist of ordinal data and therefore were analyzed by nonparametric Kruskal-Wallis tests, followed by Dunn’s post hoc tests. All other measures were analyzed by one-way ANOVA followed by Holm-Sidak’s post hoc analyses. Details of the statistical tests are contained within the figure legends, along with group sizes if they differ from the full experimental group size. Where reported in the text, the mean (*M*) and standard deviation (*SD*) are given in parenthesis. Statistical significance was set at p < 0.05 and graphs show group means with error bars representing the standard error of the mean (SEM). Except where otherwise noted, Graphpad Prism software version 9.2.0 was used for statistical analyses and figure generation. The full dataset used to generate the statistical analyses and graphs for each figure is included in **S1 File**.

## Results

### MW151 has little to no effect on observational or intrinsic behavioral changes in the MD mice

The APPswe/PS1dE9 mice on HHcy diet (MD mice) lost significant weight after 8 weeks versus WT ctrl, consistent with the expected effects of HHcy induction [19, 21], with no differences between the groups assigned to receive saline (*M* = -16.4%, *SD* = 6.4) or MW151 (*M* = -16.1%, *SD* = 4.3) (**Fig 1A**). The weight regained after the recovery period was similarly comparable between the saline (*M* = 0.2%, *SD* = 6.2) and MW151 MD groups (*M* = 0.4%, *SD* = 5.9). By the time of sacrifice, there was no difference in weight change between the WT ctrl (*M* = -1.6%, *SD* = 5.0), MD saline (*M* = -3.8%, *SD* = 7.2), or MD MW151 groups (*M* = -2.2%, *SD* = 7.4). Plasma homocysteine levels were below the limit of detection (< 5 μmol/l) in all the samples tested, indicating full recovery of the acute HHcy by the time of sacrifice. In terms of the non-cognitive aspects of the behavioral battery, no differences were found in the nesting (**Fig 1B**) or marble burying assays (**Fig 1C**) for intrinsic behaviors. For the frailty index, the MD groups had higher frailty scores than the WT ctrl group, but only the comparison between MW151-treated MD mice and WT ctrl reached statistical significance (see **S2 Table** for grading criteria and definitions) (**Fig 1D**), with no differences in core body temperature between WT control (*M* = 37.2°C, *SD* = 0.7), WT HHcy (*M* = 36.9°C, *SD* = 0.9), MD saline (*M* = 37.3°C, *SD* = 0.7), or MD MW151 (*M* = 37.2°C, *SD* = 0.4) groups (one-way ANOVA, *F*(3, 51) = 1.006, *p* = 0.398). General locomotor and anxiety-type behavior was assessed in a 15-minute OF assay (**Fig 1E**), with an increase in average speed observed in the MD saline (*M* = 7.8 cm/s, *SD* = 2.1) and MD MW151 (*M* = 7.2 cm/s, *SD* = 1.6) groups relative to WT ctrl (*M* = 5.6 cm/s, *SD* = 0.6) (**Fig 1E** left panel). Similarly, time spent in the center of the arena was reduced in both MD saline (*M* = 137.6 s, *SD* = 37.9) and MD MW151 mice (*M* = 148.9 s, *SD* = 38.2 s) versus WT ctrl (*M* = 188.6 s, *SD* = 48.3) indicative of an anxiogenic phenotype (**Fig 1E** right panel). MW151 had no effect on either of these parameters.

### MW151 shows a trend toward effects on RAWM performance

Results of hippocampal-dependent memory testing in the MD mice are summarized in **Fig 2**. WT ctrl mice spent significantly more time with the object in the novel versus familiar location, but the MD saline mice did not spend significantly more time with the object in either location, nor did those treated with MW151 (**Fig 2A**). Similarly, MD saline mice were found to be impaired in the RAWM task, where they committed significantly more errors on day 2 (*M* = 3.3, *SD* = 1.0) and day 3 (*M* = 2.8, *SD* = 1.2) relative to the WT ctrl group (*M* = 2.6, *SD* = 0.7 and *M* = 1.6, *SD* = 0.7 on days 2 and 3, respectively) (**Fig 2B**). In contrast to the OLT, MW151 treatment may have improved the performance of the MD mice in the RAWM, where the MD MW151 did not commit significantly more errors than WT ctrl on either day 2 (*M* = 2.7, *SD* = 1.0) or day 3 (*M* = 2.1, *SD* = 0.7) of testing; however, the reduced mean errors of the MD MW151 versus MD saline mice did not reach statistical significance. Additionally, no group differences were found in OP final block average latency to platform nor overall average swim speed, indicating that increased errors may be primarily attributable to memory deficits rather than alterations in motivation, vision, or swimming capability (**Fig 2C**). Taken together, these data indicate that at least some aspects of hippocampal-dependent memory function may be improved by MW151 in this MD model; however, replication with larger group sizes will be necessary to confirm this trend.

### MW151 normalizes hippocampal levels of NAA and NAAG, with no effect on CBF

**Fig 3:**
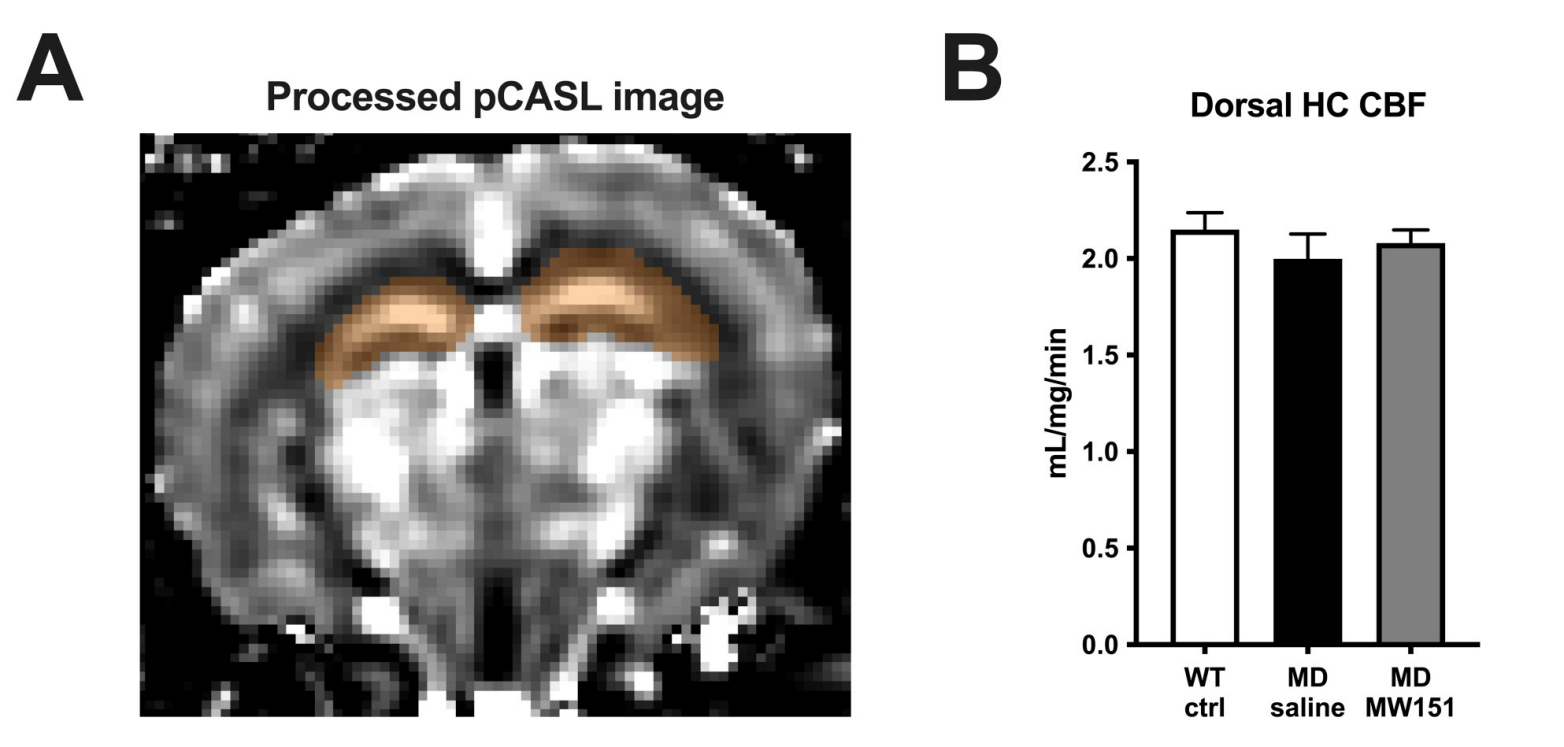
Hippocampal blood flow is unimpaired in MD mice after HHcy recovery and unaltered by treatment with MW151. (**A**) Perfusion-weighted pCASL image is shown, with the bilateral dorsal hippocampal region of interest colored in brown. (**B**) Quantification of group averages showed no difference between any of the groups by one-way ANOVA (*F*(2, 27) = 0.602, *p* = 0.555).

**Fig 4:**
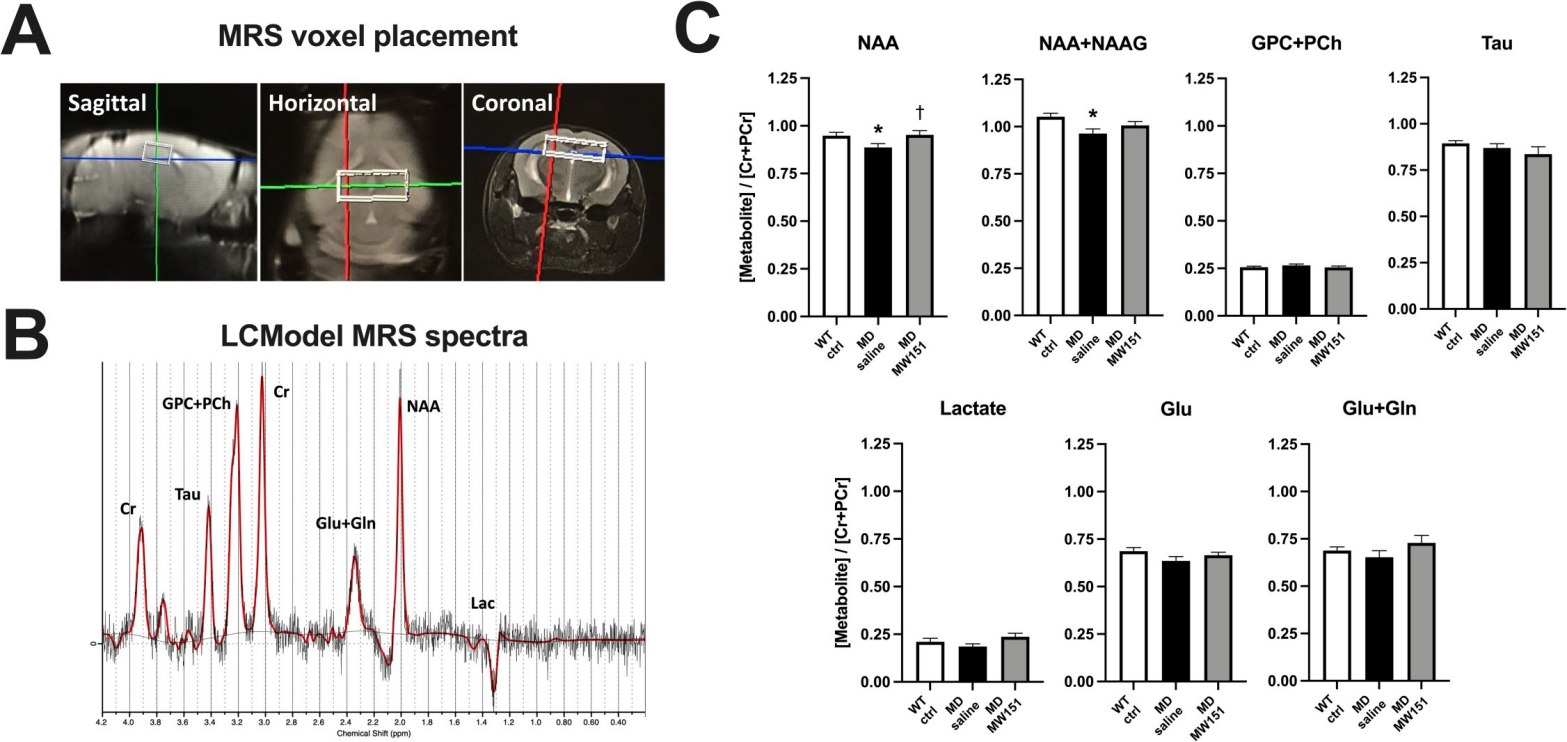
Hippocampal NAA/creatine ratio is fully restored in MD mice after treatment with MW151. (**A**) Example voxel placement over the bilateral hippocampus is shown from sagittal, horizontal, and coronal perspectives. (**B**) An example of the MRS spectra output from LCmodel is shown. (**C**) Metabolites expressed as a ratio to total creatine concentration (Cr+PCr) are shown. Results of one-way ANOVAs indicated significant differences in average NAA ratio (*F*(2, 27) = 3.550, *p* = .0428) and NAA+NAAG ratio (*F*(2, 27) = 4.4189, *p* = 0.026), with Holm-Sidak post hoc tests showing decreases in the MD saline but not MD MW151 groups, *p < .05 versus WT ctrl, †p < .05 versus MD saline.

A separate cohort of mice comprised of the three primary experimental groups (WT ctrl, MD saline, and MD MW151) were treated in the same manner as above but underwent measurement of hippocampal CBF by pCASL (**Fig 3**), followed immediately by hippocampal metabolite measurement by MRS (**Fig 4**). The processed pCASL image is shown in **Fig 3A**, with the bilateral dorsal hippocampal region of interest colored in brown. As shown in **Fig 3B**, no differences in hippocampal CBF were detectable in MD saline mice (*M* = 2.00 mL/mg/min, *SD* = 0.41) versus WT ctrl (*M* = 2.15 mL/mg/min, *SD* = 0.28), nor did MW151 treatment have an effect (*M* = 2.08 mL/mg/min, *SD* = 0.22). Immediately after pCASL, MRS was performed in the bilateral dorsal hippocampus with the voxel placement shown in **Fig 4A**. An example of the LCModel-processed spectra is shown in **Fig 4B**. Metabolite concentrations, expressed as ratios divided by the total creatine (Cr + PCr) concentration, are shown in **Fig 4C**. In the MD saline group, the NAA (*M* = 0.887, *SD* = 0.063) and the combined NAA+NAAG ratios (*M* = 0.963, *SD* = 0.079) were found to be significantly reduced compared to the corresponding WT ctrl NAA (*M* = 0.949, *SD* = 0.054) and NAA+NAAG ratios (*M* = 1.052, *SD* = 0.061). Treatment of the MD mice with MW151 normalized both the NAA (*M* = 0.953, *SD* = 0.068) and the NAA+NAAG ratios (M = 1.006, SD = 0.065). No significant differences were seen among the groups for any of the other metabolites that met inclusion criteria (GPC+PCh, Tau, Lactate, Glu, or Glu+Gln).

### Functional effects of MW151 administration are associated with known anti-inflammatory activities and no effect on amyloid burden

**Fig 5:**
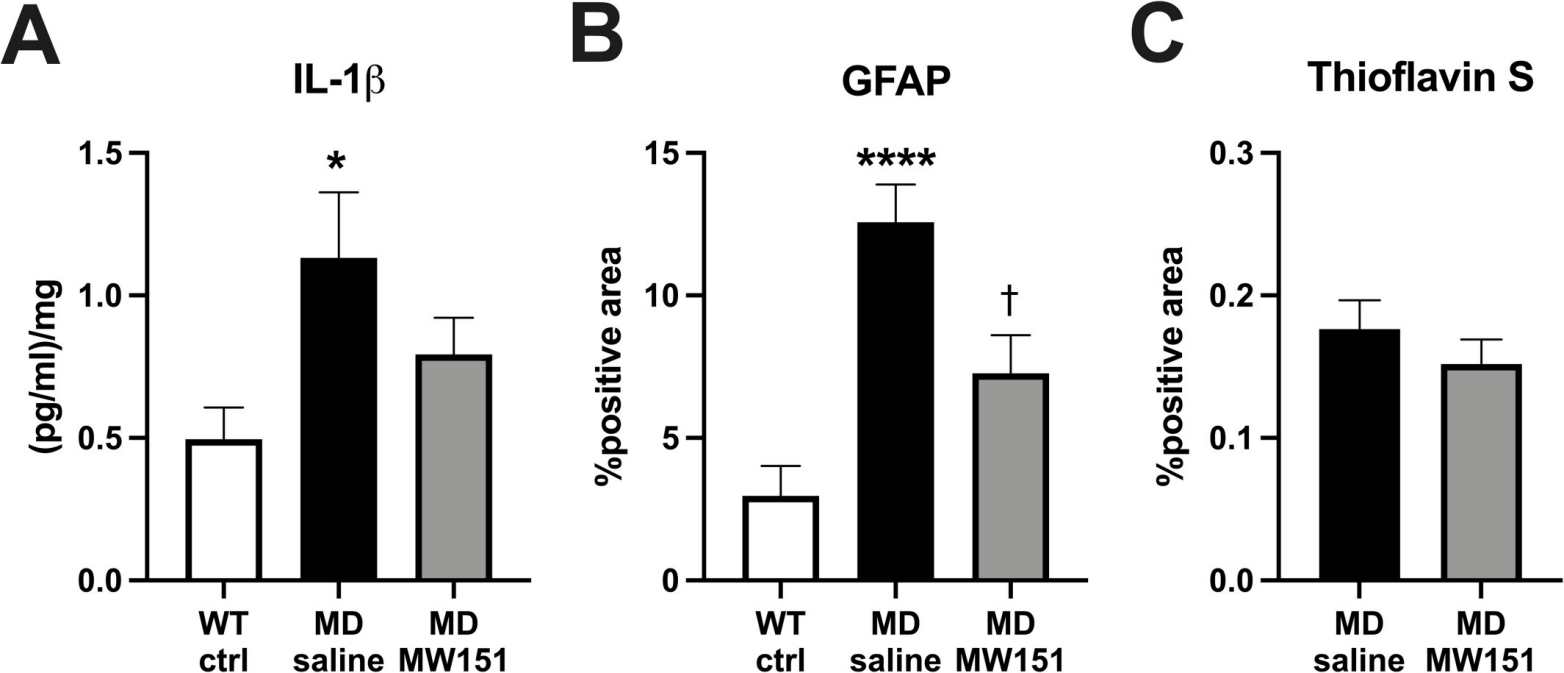
MW151 treatment reduces hippocampal IL-1β and GFAP elevations in the MD mice, with no effect on Thioflavin S positive amyloid plaque burden. (**A**) MD saline mice (n = 12) had significantly greater IL-1β levels in the hippocampus versus WT ctrl (n = 13), an effect not observed in the MD MW151 mice. One-way ANOVA (F(2, 36) = 3.822, p = 0.0312), *p < .05 versus WT ctrl, Holm-Sidak post hoc. (**B**) Hippocampal GFAP positivity is increased in the MD saline mice (n = 10) versus WT ctrl (n = 12), but not MD MW151 mice (n = 10). One-way ANOVA (F(2, 29) = 15.66, p < .0001), ****p < .0001 versus WT ctrl, †p < .05 versus MD saline, Holm-Sidak post hocs. **(C)** No differences were seen in amyloid plaque burden, as assessed by Thioflavin S staining, between MD saline and MD MW151 mice.

Previous studies in mouse amyloid models of AD have shown that MW151 administration causes a reduction in brain levels of the proinflammatory cytokine IL-1® and astrocyte activation as quantified by GFAP positivity, with no effect on underlying plaque burden [7, 16]. We confirmed the presence of this signature in the MD model by pathological assessment of these parameters within the hippocampus of mice from the behavioral battery experiment (**Fig 5**). As expected, hippocampal IL-1® is elevated in the MD saline group (*M* = 1.13 pg/ml/mg, *SD* = 0.80) compared to WT ctrl (*M* = 0.50 pg/ml/mg, *SD* = 0.40), becoming statistically indistinguishable from controls in the MD MW151 group (M = 0.79 pg/ml/mg, SD = 0.48) (**Fig 5A**). Hippocampal GFAP percent positivity follows a similar pattern (**Fig 5B**), with an increase in the MD saline mice (*M* = 12.57%, *SD* = 4.18) relative to WT ctrl (*M* = 2.96%, *SD* = 3.66), decreased by MW151 treatment (*M* = 6.66%, *SD* = 4.49). No effect on plaque burden as measured by Thioflavin S-positivity was seen in the MD MW151 mice compared to the MD saline mice (**Fig 5C**). IL-6, TNFα, and CXCL1 were not detectably altered in the hippocampus of the MD mice, nor modulated by MW151 treatment (**S3 Fig**). That these were not detectably elevated may be due to some combination of: complex interactive effects of amyloid and HHcy on neuroinflammatory markers [21], the time point of analysis, the region of analysis, or the measurement of protein rather than mRNA levels.

## Discussion

We found that therapeutic administration of MW151 partially reduces hippocampal-dependent cognitive deficits in a mixed dementia model, that this trend toward a functional effect corresponds with a restoration of the neuronal integrity marker NAA and the neuropeptide NAAG, and that these changes are associated with the established anti-inflammatory signature of this drug. These findings provide further support for the potential clinical utility of MW151, by showing activity in a preclinical model of comorbid AD and vascular pathology, a mixed pathology very commonly seen in AD patients.

The strain of APPswe/PS1dE9 mice used in this study was chosen because of its well-characterized and progressive accumulation of plaque pathology, neuroinflammatory changes, and eventual cognitive dysfunction [25–27]. Interestingly, this strain has been reported to display at least some types of tau pathology at 18 months of age [36]. This model is therefore one based on the conceptualization of AD as an amyloid-driven tauopathy, and the age of the mice in this study should be considered representative of a fairly early stage in the disease process. HHcy was chosen to induce vascular damage not only because it recapitulates a broad array of the vascular pathologies seen in patients (*e*.*g*. microhemorrhages, CBF deficits, small vessel pathology) but because it is itself a prevalent independent risk factor for AD [37]. This was weighed against potential concerns about the model given its severity, broad systemic effects that might contribute to off-target pathologies and/or interfere with drug metabolism, as well as the fact that any patient presenting with severe HHcy secondary to B-vitamin deficiency would first be treated with B-vitamin supplementation. To mitigate these concerns, we incorporated a 2-week dietary recovery period in which mice were returned to standard rodent chow with adequate B-vitamins and normal methionine levels. Importantly, we verified that despite normalization of plasma homocysteine and partial recovery of the diet-induced weight loss, transient 8-week HHcy is sufficient to produce microhemorrhages and persistent deficits in both short-term hippocampal-dependent spatial memory as well as longer term spatial learning (**S1 and S2 Figs**). It is important to note that although we find evidence of vascular injury alongside the persistent cognitive deficits, the Prussian blue stain does not differentiate current from previous vascular injury. Therefore, we cannot definitively conclude that vascular damage is causative of the persistent cognitive deficits and caution must be used in interpreting the HHcy-associated injury as strictly or primarily vascular in nature. Other mechanisms (e.g. epigenetic changes) may also play a role. Nonetheless, these data appear consistent with epidemiological literature regarding vitamin supplementation for HHcy. Meta-analyses of this type of intervention clearly shows it to be effective in lowering homocysteine levels, with apparently little to no cognitive benefit [38, 39]. Although there is some dispute about the validity of the design of these trials [40], an exploration of the underlying pathological explanations for persistent cognitive deficits following transient HHcy is an interesting and relevant direction for future work.

Considering that MW151 would most likely be administered initially in a therapeutic (as opposed to preventative) paradigm, we employed this strategy in the MD mice. Dosing occurred subsequent to the development of amyloid and HHcy pathologies, and all functional testing was performed with mice “on drug” in an attempt to mirror the potential future clinical situation. We also included several non-cognitive phenotyping assays to explore changes in gross physiology, general motoric function, anxiety-like behaviors, and intrinsic behaviors. As has been previously reported for APPswe/PS1de9 mice, hyperlocomotive and anxiogenic phenotypes [41] were detected in the OF, as well as overall deficits in nest-building [42]. The latter did not reach significance in post-hoc testing, potentially indicative of a lack of power for this test. We also found an increase in aging-associated physiological changes in the MD mice according to the frailty index, but the data reached significance only in the MW151-treated MD mice. Presumably this is due to a reduction in variability in this group relative to the MD saline group. No change was observed in burrowing behavior, as represented by the marble burying task. Assuming that the OF behaviors represent meaningful aspects of the human disease, these data imply that MW151 by itself may not provide much benefit toward the non-cognitive aspects of AD.

In terms of the spatial memory deficits, MW151 had mixed effects. While the compound did not rescue deficits in the OLT, it may have modestly improved the RAWM performance of MD mice. Although a replication study with larger group sizes is required to confirm this possibility, there are plausible mechanisms to explain the effects. Given that pathologically increased IL-1β in the hippocampus can interfere with some forms of hippocampal-dependent memory (for review see [43]), it is plausible that the memory benefits are linked to its reduction. It is unclear why this would not be true for the OLT as well, but there are at least two possibilities. Firstly, due to the limitation of the “on drug” design of this study, it may simply be because there were fewer doses of MW151 given at the time of the OLT versus RAWM. Perhaps a benefit in OLT would have been apparent if MW151 was administered for a longer period of time prior to testing. This can be addressed by additional studies that parse the time course of anti-inflammatory versus behavioral changes resulting from MW151 treatment. Alternatively, there may be important molecular distinctions between short- and long-term hippocampal-dependent memory processes and these processes could have different sensitivities to particular alterations in the neuroinflammatory milieu. For example, IL-1β can reduce expression of the AMPA receptor subunit GluA1R [44], which has been shown to have opposing roles in short- versus long-term memory processes [45]. Perhaps reduction of pathologically elevated IL-1β tends to favor a recovery of longer-term learning and memory. Such mechanistic questions are outside the scope of the present translational study, but they will be important in understanding how particular anti-inflammatory effects are associated with task-specific memory processes. This understanding will be vital in tailoring appropriate therapeutic strategies. Additionally, it is important to note that MW151 was only given for a short time in the present study. That we still observed a trend toward effects on behavior could imply utility as a symptomatic treatment as well as a disease modifying one. Finally, while we report a reduction of GFAP staining as primarily a marker for the known signature of MW151 in AD models, there are likely to be additional important astrocytic effects worth characterizing more fully in future studies.

We did not detect any reduction in hippocampal blood flow in the MD mice, presumably due to the recovery of acute HHcy. Elevated homocysteine can cause acute endothelial dysfunction via impairment of nitric oxide signaling or increased production of reactive oxygen species and associated oxidative stress [46]; over time it can also cause vessel hypertrophy and arterial remodeling that increases vascular resistance [47]. Although the specific mechanisms of CBF impairment were not addressed in the present study, we interpret the data to indicate that this HHcy paradigm favors acute and transient rather than persistent mechanisms of CBF impairment. Interestingly, we did observe significant reductions in the NAA and NAA+NAAG metabolite ratios; both of which were restored upon treatment with MW151. NAA and NAAG have both been found to be reduced in the hippocampus of AD patients [48], and NAA is also reduced in the hippocampus of individuals with mild cognitive impairment [49]. NAA is considered to be a marker of neuronal integrity, and hippocampal levels of NAA have been noted to be decreased in various mouse amyloid AD models [50]. NAAG is a peptide neurotransmitter with well-characterized effects on metabotrobic glutamate receptors, and elevation of NAAG signaling has been associated with cognitive benefit [51]. Whether or not HHcy by itself causes MRS-detectable metabolic changes was not assessed in this study but would be an interesting question to answer, although in light of the CBF data it seems more likely that this is driven primarily by the amyloid pathology. Importantly, the MRS-detectable reduction in NAA is consistently observed in various brain regions of AD patients, and this may therefore represent a good candidate for a non-invasive surrogate marker of efficacy in clinical trials [52]. The restoration of hippocampal NAA and NAA+NAAG levels, alongside previous work showing a rescue of hippocampal LTP and increase in synaptic proteins with MW151 treatment [7], indicate important neuronal impacts that should be explored further. As the specific target of MW151 is unknown, we cannot determine whether the anti-inflammatory actions are causative of these neuronal effects, partial contributors, or simply act in parallel. Answering such questions and identifying the specific mechanism of action will aid in future drug development programs. Overall, our study provides further evidence that MW151 may be a useful therapeutic in the presence of multiple overlapping neuropathologies and suggests that MRS-detectable metabolic changes may be a useful surrogate marker of efficacy directly translatable to clinical settings.

## Supporting information

S1 Fig Transient HHcy induces persistent hippocampal-dependent memory deficits in WT mice. (**A**) WT mice on 8-weeks of HHcy diet lost significant weight versus those on control diet and, despite a partial recovery, remained below WT ctrl mice throughout the study. Results of two-way mixed measures ANOVA revealed a significant effect of time (*F*(1.693, 86.34) = 178.0, *p* < .0001), group (*F*(3, 51) = 13.02, *p* < .0001), and a time by group interaction (*F*(6, 102) = 24.02, *p* < .0001). ****p < .001, **p < .01, *p < .05 versus WT ctrl, Sidak’s post hoc analyses. (**B**) In the 5-minute OLT, only the WT ctrl mice spent significantly more time with the novel versus the familiar object (*T*(13) = 3.522, *p* = 0.0149) *p < .05, paired t-tests with Holm-Sidak adjustment. (**C**) Learning curves of RAWM errors by block (3 consecutive trials) are shown for WT ctrl and HHcy mice in the left panel, with RAWM errors collapsed by day for statistical analysis in the right panel. WT HHcy mice committed significantly more errors versus WT ctrl in the RAWM on day 3. Two-way mixed measures ANOVA showed a significant effect of day (*F*(1.955, 97.73) = 41.08, *p* < .0001) and group (*F*(3, 50) = 4.088, *p* = 0.0113), *p < .05, Tukey’s post-hoc tests. (**D**) Results of one-way ANOVA for average latency to platform in the final block (*F*(3, 50) = 1.076, *p* = .368) and average swim speed (*F*(3, 50) = 0.278, *p* = 0.841) indicated no statistical difference between any groups.
(DOCX)

S2 Fig Prussian blue positive objects are increased in the MD saline versus WT control groups. Remaining sections from the WT ctrl and MD saline groups that were of sufficient integrity for IHC (n = 12 WT ctrl, n = 9 MD saline) were stained with Prussian blue and counter-stained with nuclear fast red (Abcam, cat no. ab150674), according to kit manufacturer instructions. 17–26 sections were stained per mouse. The HALO classifier algorithm was trained on positive control tissue, then run on the experimental samples. A blinded investigator manually confirmed the algorithm output and divided the total number of confirmed PB-positive (PB+) objects by the number of sections analyzed. An example positive stain is shown in the left panel, and quantification in the right panel. The average number of PB+ objects per section was significantly increased in the MD saline versus WT ctrl, student’s t-test, *t*(19) = 3.744, *p* = .0014. These data are consistent with vascular injury in the MD saline model.
(DOCX)

S3 Fig Cytokine levels in the hippocampus. Hippocampal levels (mean ± SEM) of CXCL1, IL-6 and TNFα are displayed for WT, MD saline, and MD MW151 groups. No significant differences were observed between any of the groups for any of the three cytokines.
(DOCX)

S1 Table Dietary information. Nutrition and formulation of the control and HHcy-inducing diets.
(DOCX)

S2 Table Frailty assay. Frailty scoring criteria.
(DOCX)

S1 File Minimal dataset. Minimal dataset for all figures and statistical analyses in the manuscript.
(XLSX)
