# Supplementary material for: Therapeutic treatment with the anti-inflammatory drug candidate MW151 may partially reduce memory impairment and normalizes hippocampal metabolic markers in a mouse model of comorbid amyloid and vascular pathology
Source: PLoS One. 2022 Jan 26;17(1):e0262474. doi: 10.1371/journal.pone.0262474 (PMC8791470; doi:10.1371/journal.pone.0262474)
Supplement: S1 Fig — (A) WT mice on 8-weeks of HHcy diet lost significant weight versus those on control diet and, despite a partial recovery, remained below WT ctrl mice throughout the study. Results of two-way mixed measures ANOVA revealed a significant effect of time (F(1.693, 86.34) = 178.0, p < .0001), group (F(3, 51) = 13.02, p < .0001), and a time by group interaction (F(6, 102) = 24.02, p < .0001). ****p < .001, **p < .01, *p < .05 versus WT ctrl, Sidak’s post hoc analyses. (B) In the 5-minute OLT, only the WT ctrl mice spent significantly more time with the novel versus the familiar object (T(13) = 3.522, p = 0.0149) *p < .05, paired t-tests with Holm-Sidak adjustment. (C) Learning curves of RAWM errors by block (3 consecutive trials) are shown for WT ctrl and HHcy mice in the left panel, with RAWM errors collapsed by day for statistical analysis in the right panel. WT HHcy mice committed significantly more errors versus WT ctrl in the RAWM on day 3. Two-way mixed measures ANOVA showed a significant effect of day (F(1.955, 97.73) = 41.08, p < .0001) and group (F(3, 50) = 4.088, p = 0.0113), *p < .05, Tukey’s post-hoc tests. (D) Results of one-way ANOVA for average latency to platform in the final block (F(3, 50) = 1.076, p = .368) and average swim speed (F(3, 50) = 0.278, p = 0.841) indicated no statistical difference between any groups. (DOCX) [file pone.0262474.s001.docx]

**

S2 Fig: Transient HHcy induces persistent hippocampal-dependent memory deficits in WT mice.** (**A**) WT mice on 8-weeks of HHcy diet lost significant weight versus those on control diet and, despite a partial recovery, remained below WT ctrl mice throughout the study. Results of two-way mixed measures ANOVA revealed a significant effect of time (*F*(1.693, 86.34) = 178.0, *p* < .0001), group (*F*(3, 51) = 13.02, *p* < .0001), and a time by group interaction (*F*(6, 102) = 24.02, *p* < .0001). *****p* < .001, ***p* < .01, **p* < .05 versus WT ctrl, Sidak’s post hoc analyses. (**B**) In the 5-minute OLT, only the WT ctrl mice spent significantly more time with the novel versus the familiar object (*T*(13) = 3.522, *p* = 0.0149) **p* < .05, paired t-tests with Holm-Sidak adjustment. (**C**) Learning curves of RAWM errors by block (3 consecutive trials) are shown for WT ctrl and HHcy mice in the left panel, with RAWM errors collapsed by day for statistical analysis in the right panel. WT HHcy mice committed significantly more errors versus WT ctrl in the RAWM on day 3. Two-way mixed measures ANOVA showed a significant effect of day (*F*(1.955, 97.73) = 41.08, *p* < .0001) and group (*F*(3, 50) = 4.088, *p* = 0.0113), **p* < .05, Tukey’s post-hoc tests. (**D**) Results of one-way ANOVA for average latency to platform in the final block (*F*(3, 50) = 1.076, *p* = .368) and average swim speed (*F*(3, 50) = 0.278, *p* = 0.841) indicated no statistical difference between any groups.
