# Supplementary material for: Therapeutic treatment with the anti-inflammatory drug candidate MW151 may partially reduce memory impairment and normalizes hippocampal metabolic markers in a mouse model of comorbid amyloid and vascular pathology
Source: PLoS One. 2022 Jan 26;17(1):e0262474. doi: 10.1371/journal.pone.0262474 (PMC8791470; doi:10.1371/journal.pone.0262474)
Supplement: S2 Fig — Remaining sections from the WT ctrl and MD saline groups that were of sufficient integrity for IHC (n = 12 WT ctrl, n = 9 MD saline) were stained with Prussian blue and counter-stained with nuclear fast red (Abcam, cat no. ab150674), according to kit manufacturer instructions. 17–26 sections were stained per mouse. The HALO classifier algorithm was trained on positive control tissue, then run on the experimental samples. A blinded investigator manually confirmed the algorithm output and divided the total number of confirmed PB-positive (PB+) objects by the number of sections analyzed. An example positive stain is shown in the left panel, and quantification in the right panel. The average number of PB+ objects per section was significantly increased in the MD saline versus WT ctrl, student’s t-test, t(19) = 3.744, p = .0014. These data are consistent with vascular injury in the MD saline model. (DOCX) [file pone.0262474.s002.docx]

**S3 Fig: Prussian blue positive objects are increased in the MD saline versus WT control groups.** Remaining sections from the WT ctrl and MD saline groups that were of sufficient integrity for IHC (n = 12 WT ctrl, n = 9 MD saline) were stained with Prussian blue and counter-stained with nuclear fast red (Abcam, cat no. ab150674), according to kit manufacturer instructions. 17-26 sections were stained per mouse. The HALO classifier algorithm was trained on positive control tissue, then run on the experimental samples. A blinded investigator manually confirmed the algorithm output and divided the total number of confirmed PB-positive (PB+) objects by the number of sections analyzed. An example positive stain is shown in the left panel, and quantification in the right panel. The average number of PB+ objects per section was significantly increased in the MD saline versus WT ctrl, student’s t-test, *t*(19) = 3.744, *p* = .0014. These data are consistent with vascular injury in the MD saline model.
