# Supplementary material for: Therapeutic treatment with the anti-inflammatory drug candidate MW151 may partially reduce memory impairment and normalizes hippocampal metabolic markers in a mouse model of comorbid amyloid and vascular pathology
Source: PLoS One. 2022 Jan 26;17(1):e0262474. doi: 10.1371/journal.pone.0262474 (PMC8791470; doi:10.1371/journal.pone.0262474)
Supplement: S3 Fig — Hippocampal levels (mean ± SEM) of CXCL1, IL-6 and TNFα are displayed for WT, MD saline, and MD MW151 groups. No significant differences were observed between any of the groups for any of the three cytokines. (DOCX) [file pone.0262474.s003.docx]

**S5 Fig. Cytokine levels in the hippocampus.** Hippocampal mean levels of CXCL1, IL-6 and TNFα are displayed for WT, MD saline, and MD MW151 groups. No significant differences were observed between any of the groups for any of the three cytokines.
