# Supplementary material for: Therapeutic treatment with the anti-inflammatory drug candidate MW151 may partially reduce memory impairment and normalizes hippocampal metabolic markers in a mouse model of comorbid amyloid and vascular pathology
Source: PLoS One. 2022 Jan 26;17(1):e0262474. doi: 10.1371/journal.pone.0262474 (PMC8791470; doi:10.1371/journal.pone.0262474)
Supplement: S1 Table — Nutrition and formulation of the control and HHcy-inducing diets. (DOCX) [file pone.0262474.s004.docx]

|  | **TD.01636 control** | **TD.97345 HHcy** |
| --- | --- | --- |
|  | | |
| Nutrition information | % kcal from | % kcal from |
| Protein | 18.5 | 19 |
| Carbohydrate | 65.1 | 64.7 |
| Fat | 16.3 | 16.3 |
| Kcal/g | 3.9 | 3.9 |
|  | | |
| Formulation | (g/kg) | (g/kg) |
| Casein | 195 | 195 |
| DL-Methionine | 3 | 7.7 |
| Sucrose | 340.486 | 345.567 |
| Corn starch | 300 | 300 |
| Soybean oil | 70 | 70 |
| Cellulose | 40 | 40 |
| Mineral mix AIN-93G-MX (94046) | 35 | 35 |
| Calcium phosphate, dibasic | 4 | 4 |
| TBHQ, antioxidant | 0.014 | 0.014 |
| Vitamin Mix AIN-93-VX (94047) | 10 | - |
| Choline Bitartrate | - | 2.5 |
| Niacin | - | 0.03 |
| Calcium Pantothenate | - | 0.016 |
| Thiamin (81%) | - | 0.006 |
| Riboflavin | - | 0.006 |
| Biotin | - | 0.0002 |
| Vitamin E (500 IU/g) | - | 0.15 |
| Vitamin A (500,000 IU/g) | - | 0.008 |
| Vitamin D3 (500,000 IU/G) | - | 0.002 |
| Vitamin K1 | - | 0.0008 |

**S1 Table: Dietary information.** Nutrition and formulation of the control and HHcy-inducing diets.
