# Supplementary material for: Therapeutic treatment with the anti-inflammatory drug candidate MW151 may partially reduce memory impairment and normalizes hippocampal metabolic markers in a mouse model of comorbid amyloid and vascular pathology
Source: PLoS One. 2022 Jan 26;17(1):e0262474. doi: 10.1371/journal.pone.0262474 (PMC8791470; doi:10.1371/journal.pone.0262474)
Supplement: S2 Table — Frailty scoring criteria. (DOCX) [file pone.0262474.s005.docx]

**S4 Table. Frailty Assay.** Frailty scoring criteria.
